# First Report of *Fusarium proliferatum* Infection in Pods of Four-Seeded Vetch and Its Relationships with Plants

**DOI:** 10.3390/plants14101480

**Published:** 2025-05-15

**Authors:** Kexin Shi, Jingxuan Xu, Hongji Wang, Xiaoliang Xue, Zihan Xie, Yuzhu Han

**Affiliations:** 1 College of Animal Science and Technology, Southwest University, Chongqing 402460, China; skx5250@163.com (K.S.); xujingxuan0128@163.com (J.X.); whongji123@163.com (H.W.); xiaoliang2007@outlook.com (X.X.); gbjrsk@163.com (Z.X.); 2 Chongqing University Herbivore Engineering Research Center, Chongqing 402460, China

**Keywords:** *Vicia tetrasperma*, *Fusarium proliferatum*, biological characteristics, biological control, metabolomics analysis

## Abstract

*Vicia* species are of great value in ecological restoration, soil improvement, and the development of a forage resource. In 2024, a novel pod disease affecting four-seeded vetches (*Vicia tetrasperma*) emerged in Rongchang District, China, leading to severe yield loss. After obtaining the main pathogenic strain, FVS1, through the tissue isolation method, which was verified according to Koch’s postulates, and by combining morphological characteristics with multigene phylogenetic analysis, FVS1 was identified as *Fusarium proliferatum*. The biological properties indicated that the most suitable culture medium of the fungus was oatmeal agar (OA), with the optimum growth temperature 25 °C and the lethal temperature being 35 °C. FVS1 exhibited insensitivity within a pH range of 7 to 9, as well as high adaptability to variations in light duration. To elucidate the physiological and biochemical changes in four-seeded vetches in response to FVS1 infection, non-targeted metabolomics analysis identified 379 differential metabolites, mainly comprising organic acids and derivatives, lipids and lipid-like molecules, and phenylpropanoids and polyketides. The results demonstrated that *F. proliferatum* primarily induced the disease by influencing alterations in the secondary metabolites associated with amino acid metabolism, lipid metabolism, and flavonoid biosynthesis. Four-seeded vetches improved tolerance to the fungus by accumulating histidine, aspartic acid, arginosuccinate, ethanolamine, glycerophosphocholine, naringenin, and catechin. *Trichoderma harzianum* (M3) had the best control effectiveness, and the inhibition rate was 60.68%. This study, for the first time, revealed that *F. proliferatum* caused a pod disease in four-seeded vetches. We analyzed the mechanism of plant–pathogen interaction and screened potential biocontrol strains, providing a theoretical basis for regional disease management.

## 1. Introduction

The genus *Fusarium* is one of the most significant plant pathogenic fungal genera globally over 330 species [1]. In addition, this genus exhibits significant ecological diversity, with its members functioning as saprophytic fungi, endophytic fungi, and plant pathogens, depending on the environmental context [2]. *Fusarium* species are known to produce mycotoxins, including fumonisins, zearalenone, and fusaproliferin [3]. These mycotoxins not only severely imperil food safety but also result in serious health hazards to animals and humans [4]. Recently, in the realm of global agricultural production, diseases attributed to the *Fusarium* genus are notably prevalent and significantly detrimental. In Fujian, China, *Fusarium proliferatum* has been proven to trigger fruit rot in peaches (*Prunus persica* L.), leading to severe losses in the local peach growing industry [5]. Postharvest fruit rot in the thin-skinned banana “Lady Finger” caused by *Fusarium sacchari* has been reported in Italy [6]. Wang et al. discovered that *Fusarium meridionale* caused stalk rot in perennial ryegrass (*Lolium perenne*) [7].

**Figure 1:**
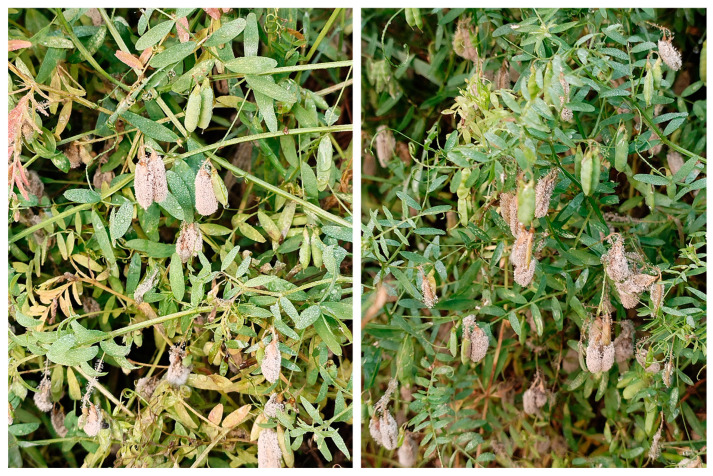
Infected plants in the field.

The four-seeded vetch (*Vicia tetrasperma*) is an annual herbaceous plant belonging to the *Vicia* genus in the Fabaceae family [8]. It is widely distributed across various regions in the Americas, Africa, and Asia [8]. Plants of the *Vicia* genus can be used as forages, green manures, and ornamental plants, which are drought- and cold-tolerant [9,10,11]. The four-seeded vetch, a member of the *Vicia* genus, holds significant research value. Currently, both domestic and international studies on the four-seeded vetch primarily concentrate on species classification, genome sequencing, and germplasm development; however, gaps remain in the exploration of the occurrence and management of diseases in four-seeded vetches, as well as the physiological and biochemical characteristics of the pathogenic fungi [12,13,14,15,16]. In 2024, we found a disease that specifically infected the pods of four-seeded vetches, exhibiting an incidence rate of 92%. The early symptoms of the disease included the emergence of small brown lesions, accompanied by mycelial proliferation. Subsequently, the lesions gradually expanded, and the surface of the pods was covered with a large amount of mycelium, ultimately leading to wilting (Figure 1).

To investigate the causes of the disease and to develop effective control strategies, we employed tissue isolation and the pin prick method of inoculation to-verify the pathogenicity of the pathogenic fungus in accordance with Koch’s postulates. We compared the morphological characteristics of the pathogen and conducted multigene phylogenetic analysis to clarify its classification status and further explore its biological characteristics. Potential biocontrol strains were screened; then, non-targeted metabolomics techniques, as well as liquid chromatography–tandem mass spectrometry (LS-MS/MS), were integrated to decipher the interactions between the pathogenic fungus and plants. This study provides a theoretical basis for the effective control of diseases affecting four-seeded vetches and serves as an important reference for understanding the relationship between four-seeded vetches and the pathogen.

## 2. Results

### 2.1. Isolation and Pathogenicity Determination of the Pathogenic Fungus

**Figure 2:**
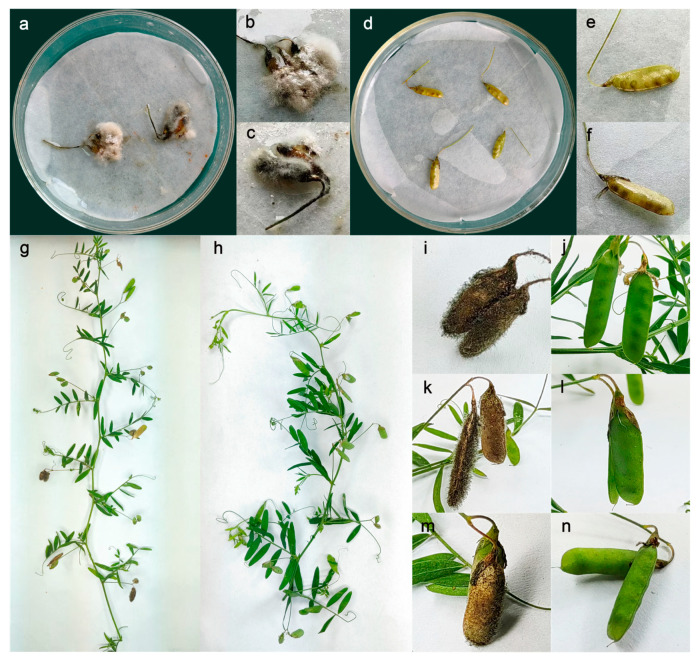
Symptoms of the pods of four-seeded vetches inoculated with FVS1 and healthy controls: (**a**–**c**) FVS1 was inoculated back onto the detached pods; (**d**–**f**) healthy, detached pods; (**g**) FVS1 was inoculated back to the four-seeded vetch plant; (**h**) healthy four-seeded vetch plant; (**i**,**k**,**m**) FVS1 was inoculated back to the pods on intact plant; (**j**,**l**,**n**) healthy pods on intact plant.

The isolation of pathogens from the pods of four-seeded vetches presenting typical symptoms, combined with morphological characterization and multigene phylogenetic analysis, revealed that the isolation rate of *F*. *proliferatum* reached 95%. A representative strain, FVS1, was selected and inoculated onto punctured healthy pods to test for pathogenicity. The experimental results showed that the FVS1 isolates were potently pathogenic, and the typical symptoms could be observed on detached pods and intact plants two days later (Figure 2), with an incidence rate of 100%. The pathogenic fungus re-isolated from the diseased pods exhibited the same morphological characteristics as FVS1.

### 2.2. Morphological Identification

**Figure 3:**
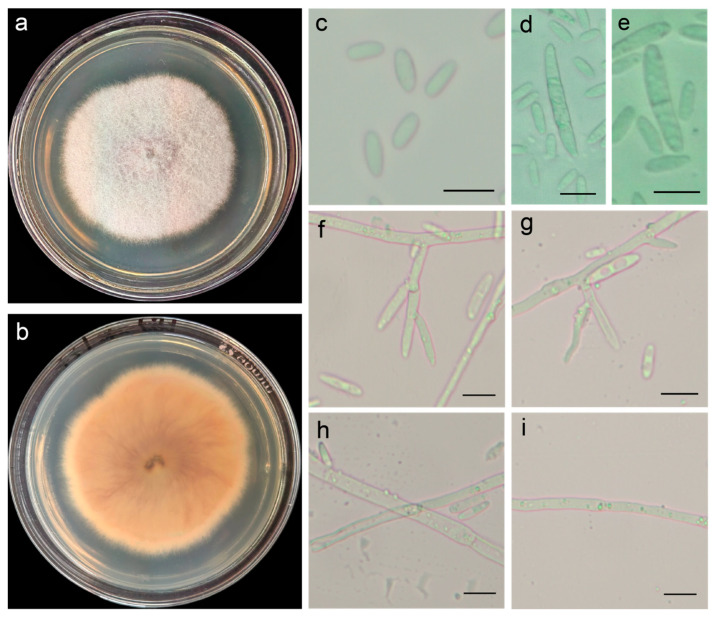
The morphology of FVS1 grown on potato dextrose agar (PDA): (**a**,**b**) the front and back morphology of FVS1 cultured for 7 d; (**c**–**e**) microconidia and macroconidia; (**f**,**g**) conidiophores and conidia; (**h**,**i**) mycelia. Gauge = 10 µM.

The pathogenic fungus, incubated at 28 °C for 7 d on potato dextrose agar (PDA), formed colonies with raised surfaces and smooth edges; the aerial hyphae were white, downy, and dense. The back of the matrix was initially pink, gradually turning purplish red (Figure 3a,b). Microscopic observation showed that the microconidia were transparent, ovoid, or oblong (6–15 μm × 2–3 μm); and the macroconidia were transparent, sickle-shaped, or linear, with 1–2 septa (10–32 μm × 3–5 μm) (Figure 3c–e). The conidiophores were straight or curved (Figure 3f,g), and the mycelia were dendritic (Figure 3h,i). The morphological characteristics of FVS1 were similar to those of *F. proliferatum* [17,18]. According to the colony culture, spore, and mycelium characteristics, along with color and size, the test strain was identified as *F. proliferatum*.

### 2.3. Phylogenetic Tree Analysis

**Figure 4:**
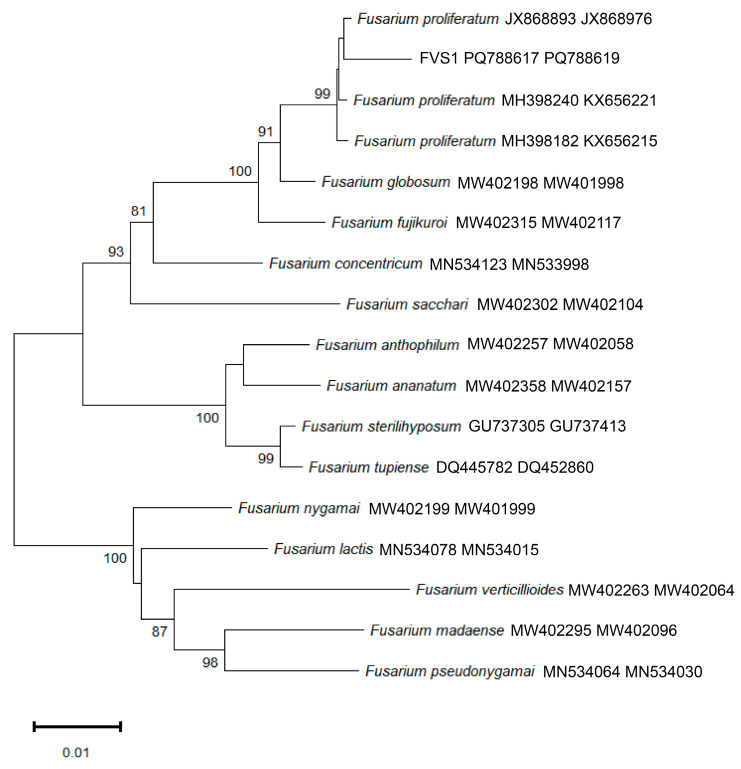
A phylogenetic tree of FVS1, constructed via the neighbor-joining method, based on *p*-distance and the analyses of multiple gene sequences (BT, EF).

To identify the obtained strains, all sequences were analyzed using BLAST 1.4.0 to retrieve homologous sequences with high similarity (Table 1). The ITS, ACT, BT, and EF gene sequences of the test strain were deposited in the National Center for Biotechnology Information (NCBI), yielding accession numbers PQ772089, PQ788618, PQ788617, and PQ788619. Phylogenetic analysis was performed using MEGA11 with BT and EF gene sequences. The resulting tree (Figure 4) based on a multilocus dataset showed that *F. proliferatum* and FVS1 clustered on the same branch, which was supported by bootstrap values of 99%. According to these findings, it was confirmed that FVS1 was *F. proliferatum*.

### 2.4. Effect of Different Media on the Colony Growth of FVS1 Strain

**Figure 5:**
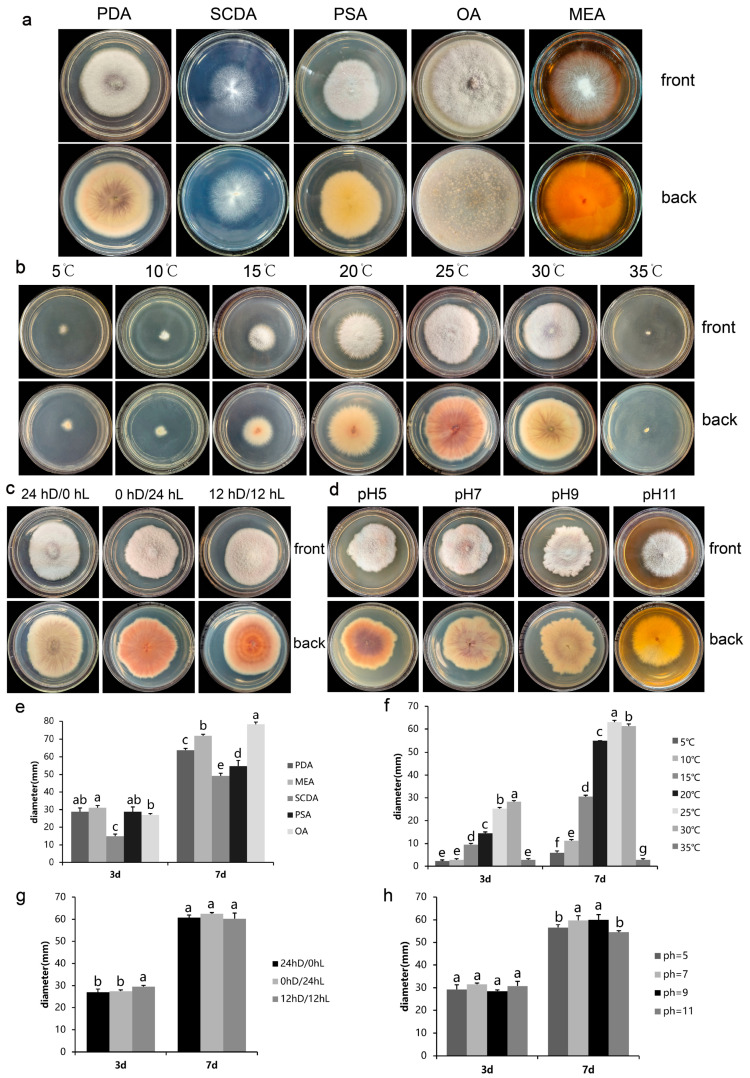
The morphological characteristics of FVS1 cultured for 7 d and the average colony diameter cultured for 3 d and 7 d under different conditions: (**a**,**e**) different media; (**b**,**f**) different temperature conditions; (**c**,**g**) different light durations; (**d**,**h**) different pH conditions. The same lowercase letters mean that the difference is not significant (*p* > 0.05); different lowercase letters indicate significant differences (*p* < 0.05).

There were significant differences in the colony diameters of FVS1 cultured on five different media for 7 d (*p* < 0.05) (Figure 5a,e). The pathogenic fungus grew fastest when incubated on oatmeal agar (OA), with an average colony diameter of 78.25 mm after 7 d. The slowest growth rate was observed when cultured on salt Czapek Dox agar (SCDA), with the smallest mean colony diameter being 49.25 mm after 7 d. These data indicated that FVS1 had the highest utilization efficiency on oats. The colony morphology of the pathogenic fungus varied significantly. The colonies cultured on PDA were white, and the mycelium was villous and relatively dense, but the middle layer was slightly thinner, with a purple pigment being visible on the back of the matrix. Compared to FVS1 cultured on PDA, FVS1 cultured on potato saccharose agar (PSA) did not produce pigment. When cultured on SCDA, the mycelium was loose and prostrate. The mycelium in the middle part of the colony cultured on malt extract agar (MEA) was dense and villous, while the outer part was loose and creeping. FVS1 cultured on SCDA and MEA did not produce pigment. The mycelium of FVS1 cultured on OA was the most dense and villous, with purple pigment production. The strains produced spores in all five media (Table 2), among which FVS1 cultured on PDA produced the highest number of spores (7.5 × 10^6^ conidia/mL). In addition, FVS1 had the smallest sporulation amounts when cultured on SCDA (1.5 × 10^6^ conidia/mL).

### 2.5. Effect of Different Temperatures on the Colony Growth of FVS1 Strain

As shown in Figure 5b,f, the colony diameter of FVS1 increased with the increase in temperature under 25 °C, then decreased with the increase above 25 °C. FVS1 was viable in the temperature range of 5–30 °C. When the temperature reached 35 °C, the colony diameter did not change, and the pathogenic fungus died. The optimum temperature of FVS1 was 25 °C, and the average colony diameter cultured for 7 d was 63.00 mm, which was significantly larger than the other treatments. In addition, the colony morphology of the pathogenic fungus changed. When cultured at 5–20 °C, the colony pigmentation was less. When cultured at 25 °C, an abundant purplish-red pigment was produced. When the temperature was 30 °C, the mycelium in the middle part of FVS1 was slightly thinner and the production of pigment was reduced. In the temperature range of 5–35 °C, FVS1 sporulated only at 15 °C, 20 °C, 25 °C, and 30 °C after 7 days of incubation; it did not produce spores at 5 °C and 35 °C (Table 2). The highest sporulation yield of FVS1 was recorded at 25 °C, reaching 3.5 × 10⁶ conidia/mL. Statistically, there was no significant difference in sporulation between 25 °C and 30 °C (*p* > 0.05). Notably, the number of spores increased with increasing temperature; then, the number declined with the decreasing temperature, paralleling the changing colony diameter trend.

### 2.6. Effect of Different Photoperiod on the Colony Growth of FVS1 Strain

FVS1 could grow under three different light conditions (24 hD/0 hL, 0 hD/24 hL, and 12 hD/12 hL), with no significant differences in colony diameter (Figure 5c,g). This suggests that FVS1 is adaptable to varying photoperiods. However, distinct morphological variations were observed among the treatments. Under continuous darkness (24 hD/0 hL), the mycelium of FVS1 was generally dense, with slightly thinner central regions in the colony. Additionally, the reverse side of the matrix displayed purple pigments that were distributed radially. The mycelial growth was denser, the back of the plate was purplish red, and the pigmentation was the most in complete light (0 hD/24 hL). Under the light condition of 12 hD/12 hL, the mycelium remained dense, and the back of the matrix exhibited a ringed pattern, accompanied by purplish-red pigmentation. FVS1 was able to produce spores under all three light conditions, and the differences were not significant (Table 3).

### 2.7. Effect of pH on the Colony Growth of FVS1 Strain

FVS1 could grow normally within the pH range of 5–11, signifying the strong adaptability of this pathogenic fungus (Figure 5d,h). When the pH values were 7 and 9, the colony margins were irregular, and the colony diameters were not significantly different but were significantly larger than those at pH 5 or 11. When FVS1 was incubated on PDA plates under pH 5, the edges of the colonies were neater compared to those at pH 7 or 9. When the pH reached 11, the colony margins were the smoothest and the mycelium was the most loose. FVS1 showed relative insensitivity to pH changes within the range of 7–9. Moreover, although the growth rate of FVS1 was notably reduced, and the substrate pigmentation underwent alterations at pH 5 or 11, the growth was not completely inhibited, confirming its capacity to survive under acidic and alkaline conditions. When the pH value was 5, FVS1 had a large amount of pigmentation. The pigment in the inner ring was purplish red, the middle ring was pink, and the outer ring was yellow. A small amount of purplish-red pigment could be observed on the back matrix at pH 7 and 9. The colonies were barely pigmented when the pH value was 11. FVS1 could sporulate in the tested pH range (Table 3), with significant differences. The number of spores was the highest when the pH value was 7 (1.2 × 10^7^ conidia/mL), while it was the lowest at pH 11 (2 × 10^6^ conidia/mL).

### 2.8. Metabolomic Profiling of Four-Seeded Vetch-FVS1 Interactions

**Figure 6:**
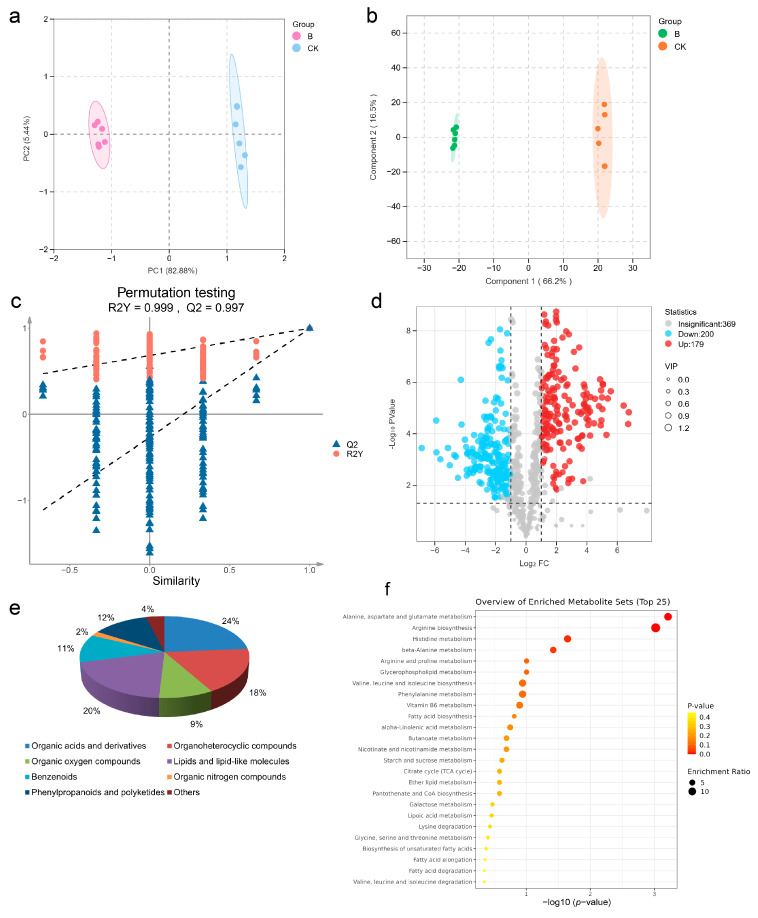
Metabolomics analysis of diseased and healthy pods of four-seeded vetches: (**a**) principal component analysis (PCA) of diseased and healthy pods of four-seed vetches; (**b**) orthogonal partial least squares discriminant analysis (OPLS-DA) of diseased and healthy pods of four-seed vetches; (**c**) OPLS-DA permutation test diagram; (**d**) volcanic plots (the red graph shows significantly up-regulated differential metabolites; the blue graph shows significantly down-regulated differential metabolites; and the gray graph shows unimportant differential metabolites); (**e**) differential metabolite classification 3D pie chart; (**f**) bubble chart. The *x*-axis represents pathway impacts, the *y*-axis represents pathway enrichment, and the color of the circle from red to yellow indicates a smaller *p*-value (*p* < 0.05). The size of the circle indicates the enrichment ratio.

**Figure 7:**
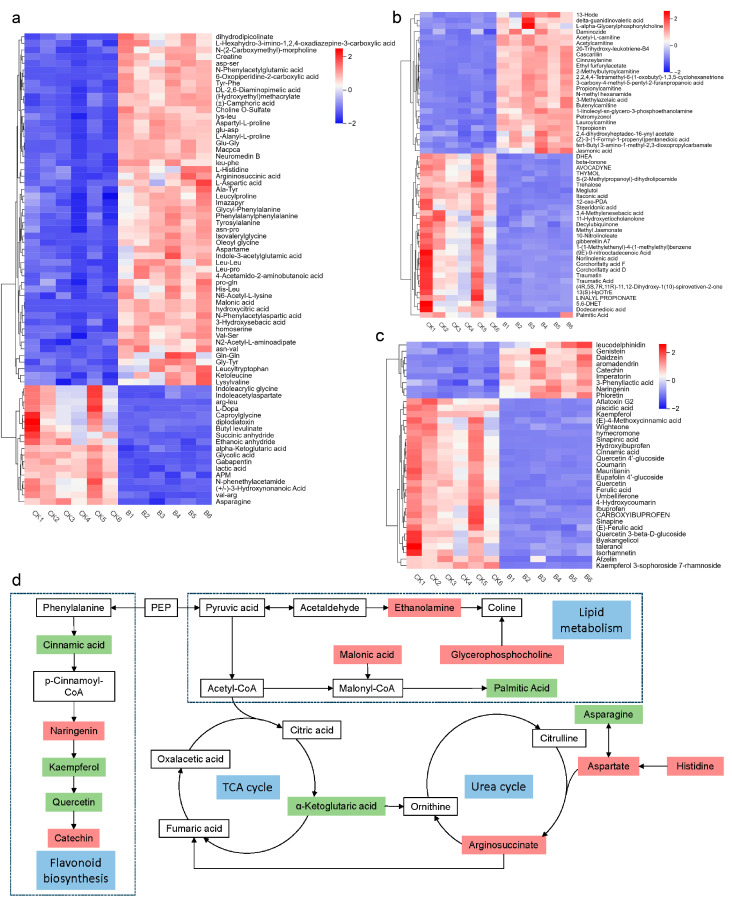
Heat maps and a pathway map: (**a**–**c**) heat maps of differential metabolites of organic acids and derivatives, lipids and lipid-like molecules, and phenylpropanes and polyketides; (**d**) pathway map. The blue boxes are used to denote metabolic pathways, the red boxes represent significantly up-regulated metabolites, and the green boxes indicate significantly down-regulated metabolites.

Metabolomics has been widely applied in studies of plant–pathogen interactions [24]. In this study, metabolomic tests were performed on diseased and healthy samples to explore the interactions between the four-seeded vetch and FVS1. The principal component analysis (PCA) score map (Figure 6a) revealed that the PC1 and the PC2 explained 82.88% and 5.44% of the variability, respectively, indicating that there were significant differences in metabolites among different groups. An orthogonal partial least squares discriminant analysis (OPLS-DA) model (Figure 6b) was established to illustrate the significant difference more intuitively between the control group (CK) and the disease group (B). The OPLS-DA model had R^2^Y = 0.999 and a Q^2^ value > 0.9, demonstrating the stability and reliability of the model (Figure 6c). To screen differential metabolites, the thresholds were set as VIP > 1.0, FC > 2, and *p* < 0.05. Through non-targeted metabolomics analysis, a total of 748 different metabolites were identified. A total of 379 metabolites with significant differences were selected, of which 200 metabolites were down-regulated and 179 metabolites were up-regulated (Figure 6d). These differential metabolites were classified into eight types, containing organic acids and derivatives, organoheterocyclic compounds, organic oxygen compounds, lipids and lipid-like molecules, benzenoids, organic nitrogen compounds, phenylpropanoids, polyketides, and other components (Figure 6e). Using MetaboAnalyst 6.0, and based on the KEGG database, metabolite pathway enrichment analysis was performed, and the bubble charts (Figure 6f) of the enriched KEGG pathways were plotted. The results suggested significant alterations in alanine, aspartate and glutamate metabolism, arginine biosynthesis, valine, leucine and isoleucine biosynthesis, histidine metabolism, and β-alanine metabolism. Heat maps (Figure 7a–c) were generated to visualize the variation in each differential metabolite. The figure shows the change patterns of three types of metabolites: organic acids and derivatives, lipids and lipid-like molecules, and phenylpropanes and polyketides. Compared with the CK group, a total of 87 metabolites were significantly up-regulated, and 74 were significantly down-regulated in the diseased samples. In addition, other functionally critical compounds in the diseased pods were significantly altered. For example, phenylacetaldehyde, carnitine, sinapyl alcohol, and leucamine in the B group were all up-regulated compared with the CK group; whereas vanillin, indole, isovanillic acid, and salicylic acid were significantly down-regulated.

After annotating the key metabolic pathways, a pathway map (Figure 7d) was constructed to elucidate the changes in metabolites in the diseased four-seeded vetches [25]. The main metabolic pathways involved lipid metabolism, tricarboxylic acid cycle (TCA cycle), amino acid metabolism, and flavonoid biosynthesis. In the glycerophospholipid metabolic pathway, acetaldehyde was transformed into ethanolamine (EA), and glycerophosphocholine (GPC) and EA were significantly up-regulated, but choline (Cho) did not accumulate. Acetaldehyde is converted into pyruvate, which is regenerated into acetyl CoA and enters the TCA cycle. Metabolites of the TCA cycle include citric acid, α-ketoglutaric acid, succinyl-CoA, fumaric acid, and oxaloacetic acid [26]. Among them, α-ketoglutaric acid was significantly down-regulated. The identified metabolites related to amino acid metabolism included aspartic acid, histidine, asparagine, glutamylglycine, etc. Compared with the CK group, aspartic acid in the B group was significantly down-regulated, histidine was significantly up-regulated, aspartic acid was significantly accumulated, and arginosuccinate was produced and involved in the urea cycle. The metabolites related to the urea cycle are ornithine, citrulline, arginosuccinate, and arginine [27]. Arginosuccinate was significantly up-regulated. On the one hand, this up-regulation stimulated the urea cycle activity and convert organic nitrogen stored in plants to inorganic nitrogen in response to pathogen stresses [28]; on the other hand, it increased the content of fumaric acid, thereby stimulating the activity of the TCA cycle. The flavonoid biosynthesis pathway exhibited a distinct response to pathogen stress, where cinnamic acid, kaempferol, coumarin, and quercetin were significantly down-regulated, while catechin and naringenin were significantly up-regulated.

### 2.9. Screening Results of Biocontrol Fungi

**Figure A1:**
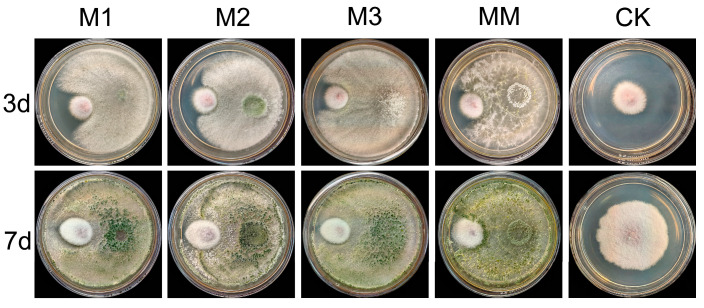
The morphological characteristics of four biocontrol strain-confronted cultures with FVS1 on PDA for 3 d and 7 d.

**Figure A2:**
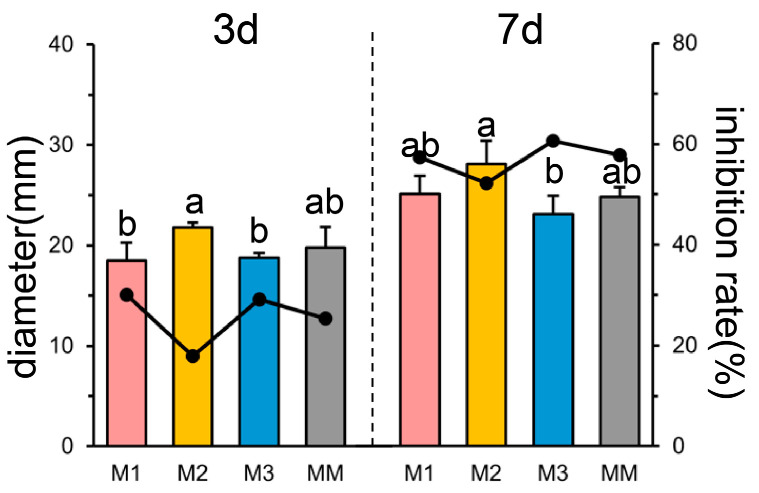
This histogram shows the average colony diameter of different biocontrol strain-confronted cultures with the FVS1 pathogen on PDA for 3 d and 7 d, and the line graph shows the inhibition rate. The same lowercase letters mean the difference is not significant (*p* > 0.05); different lowercase letters indicate significant differences (*p* < 0.05).

The results demonstrate that all four biocontrol strains of *Trichoderma harzianum* presented a good inhibitory effect on FVS1 (Appendix A, Figure A1). The inhibition rate of strain M3 was the highest, reaching 60.68%. Compared with the control group, the colony morphology of the pathogenic fungus changed. As shown in Appendix A and Figure A2, the mycelia of FVS1 were wrinkled or expanded, twisted, and deformed, and with increased branching.

## 3. Discussion

### 3.1. The Disease of the Four-Seeded Vetch Caused by F. proliferatum Was First Reported

The four-seeded vetch holds significant potential economic value. It can improve soil fertility and serves as both a forage grass and ornamental plant [29,30,31]. Therefore, studying its disease management is of great importance. In this research, the dominant pathogenic fungus *F. proliferatum* was isolated from four-seeded vetches, and the pathogenicity evaluation confirmed that *F. proliferatum* caused a serious disease in four-seeded vetches, specifically only affecting their pods. We reported for the first time that the important pathogen causing the pod disease of four-seeded vetches is *F. proliferatum*. *F. proliferatum* was a phytopathogenic fungus with a broad host range, which has been reported to induce severe disease in multiple plants. In Lucknow, India, the fungus was demonstrated to cause mango malformation disease, affecting the growth of mango (*Mangifera indica* L.) and causing 50–60% economic losses [32]. *F. proliferatum* was related to vascular wilt disease in cowpeas (*Vigna unguiculata*) and fruit rot in muskmelons (*Cucumis melo*), strawberries (*Fragaria ananassa*), and peaches [5,33,34,35]. *F. proliferatum* was the main pathogen causing leaf spot disease. For instance, Li et al. found that *F. proliferatum* could trigger leaf spot in tobacco (*Nicotiana tabacum* L.), resulting in a substantial decline in the yield [36]. Zhang et al. proved that it could infect tea (*Camellia sinensis* [L.] O. Kuntze) plants and cause them to display symptoms of leaf spot disease [37]. *F. proliferatum* has led to root rot in a variety of crops, such as peanuts (*Arachis hypogea*) and oats (*Avena sativa*) [38,39]. Additionally, it has caused Bakanae disease in Balıkesir and Canakkale, leading to a significant reduction in rice yield, and the typical symptoms included root rot [40]. Moreover, there are reports in the literature on legumes infected by *F. proliferatum*, such as faba beans (*Vicia faba*), soybeans (*Glycine max*), peas (*Pisum sativum* L.), and lentils (*Lens culinaris* L.), expressing symptoms of root rot [41,42]. Notably, it has been pointed out that *F. proliferatum* has caused the rot of soybean pods and seeds [43]. A similar phenomenon was observed on the pods of four-seeded vetches in this study, suggesting that common pathogenic mechanisms might exist in legumes. It is notable that the symptoms and severity of the disease caused by *F. proliferatum* on the four-seeded vetches are unique. For example, in mango, *F. proliferatum* mainly affects floral organs, while in four-seeded vetches, it specifically targets the pods. These differences are likely attributed to the distinct physiological characteristics and defense mechanisms of different hosts [44].

### 3.2. Control Strategy of the Pod Disease Based on Biological Characteristics of F. proliferatum

It is well known that suitable environmental conditions critically promote pathogenic fungal growth and disease progression in plants [45]. Carbon and nitrogen sources, temperature, light duration, and pH are key environmental factors. In this study, *F. proliferatum* was the primary pathogen that infected the pods of four-seed vetches. The optimum growth occurred on OA medium, while the growth rate was the slowest when the pathogen was cultured on SCDA. *F. proliferatum* exhibited the ability to grow within the tested temperature range, with an optimum temperature of 25 °C, which was consistent with the results reported by Zamir K. Punja [46]. In April 2024, when we first detected the pod disease of four-seeded vetches, the local temperature (20–30 °C) largely coincided with the appropriate growth range for *F. proliferatum*, indicating that the novel disease is likely to emerge in spring. When the temperature reached 35 °C, the pathogen died, which provided the physical control strategy of the disease. Under the three light conditions set in this study, light slightly stimulated the growth of *F. proliferatum*, yet there was no significant difference in the growth rate, which was similar to the findings reported by Francesca Fanell et al. [47]. In our research, it was found that the growth rate and sporulation amounts of *F. proliferatum* significantly decreased when the pH value was 11. 

### 3.3. Substance Regulation and Resistance Mechanism of Four-Seeded Vetches Under Stress

Metabolomics is essential for researchers in comprehending the physiological status of plants, the function of secondary metabolites, and the processes in response to biotic and abiotic stresses [48]. Bao et al. utilized non-targeted metabolomics techniques to analyze the alterations in metabolites within mulberry fruit (*Morus atropurpurea*) infected with *Ciboria shiraiana* [49]. Amino acids are involved in the synthesis of various primary and secondary metabolites [50]. They contribute to the growth and development of plants, enhance their resistance to stress, and increase overall yield [50]. In this study, over 30 metabolites related to amino acid metabolism were identified in the pods of diseased four-seeded vetches, including alanine, aspartate and glutamate metabolism, arginine biosynthesis, valine, leucine and isoleucine biosynthesis, histidine metabolism, and β-alanine metabolism. The results show that histidine and aspartic acid were significantly up-regulated, while asparagine was significantly down-regulated. Previously, Ji et al. demonstrated that increased histidine in plants could enhance the activity of antioxidant enzymes and up-regulate the expression of many stress-related genes so as to protect plants against salt stress [51]. Thus, the accumulation of histidine in four-seeded vetches is a manifestation of increased stress tolerance. Aspartic acid, as a crucial nitrogen donor, can enhance plant photosynthesis and strengthen the antioxidant defense system [52]. Down-regulation of the expression of asparagine, a form of nitrogen storage and transport, severely blocks the nitrogen metabolism network of plants [53]. When a plant experiences nitrogen deficiency, the decomposition of asparagine is intensified, and the expression of asparagine may not be high even if the aspartic acid is sufficient [54]. It is known that aspartic acid is catalyzed by asparagine synthetase (AS) to synthesize asparagine, and asparaginase (ASNase) hydrolyzes asparagine into aspartic acid and ammonia [55,56]. Therefore, if the up-regulation of aspartic acid is correlated with the down-regulation of asparagine, it is likely attributed to a decline in the content of AS and an increase in the content of ASNase in diseased plants, which may be one of the disease resistance mechanisms in four-seeded vetches.

Lipids constitute critical components of plant cell membranes and cell walls [57]. In the glycerophospholipid metabolic pathway, our results suggested that GPC and EA were significantly up-regulated, with the content of Cho having decreased. Both GPC and EA can be hydrolyzed to Cho and are related to the formation of plant cell membranes [58,59,60,61]. EA also has a defensive effect [62]. The up-regulation of GPC and EA predicted an increase in Cho content, but the results of this study indicated a decrease in the global level of Cho. According to Zhang et al. [63], it is speculated that under environmental stress, a large amount of Cho can be converted into glycine betaine (GB), a protective osmolyte. Meanwhile, to maintain membrane stability, the phosphatidylethanolamine synthesis pathway was enhanced, leading to an elevated expression of EA [57,64]. In summary, four-seeded vetches change the components of cell membrane by regulating the levels of GPC, EA, and Cho so as to meet the needs of osmoprotection and maintain normal physiological functions.

Flavonoids are pivotal secondary metabolites in plants [65]. As signal molecules, flavonoids possess potent antioxidant and antibacterial properties [65]. In this study, 36 differential metabolites of flavonoids were found, with 9 substances being significantly up-regulated, and 27 being significantly down-regulated. Among the up-regulated differential metabolites, naringin improves the resistance response in plants by interfering with the adsorption and colonization process of pathogenic fungi, regulating the antioxidant defense system and promoting synthesis of endogenous flavonoid [66,67]. Catechins have been widely proved to improve the survival of plants by scavenging reactive oxygen species and modulating redox capacity of cells [68,69,70]. Among the significantly down-regulated metabolites, cinnamic acid serves as an autotoxin. The study of Guo et al. showed that cinnamic acid promoted the occurrence of faba bean Fusarium wilt [71]. It has been pointed out that cinnamic acid secreted by the roots of peas significantly inhibits seed germination and seedling growth [72]. Overall, the four-seeded vetch may enhance stress tolerance by significantly up-regulating naringenin and catechin, while also significantly down-regulating cinnamic acid.

### 3.4. Exploring the Pathogenesis of the Pods Disease Based on Metabolomics

The findings showed that palmitic acid was significantly down-regulated in lipid metabolism, while malonic acid was significantly up-regulated. In the flavonoid synthesis pathway, kaempferol and quercetin were significantly down-regulated. Reduced palmitic acid levels compromise the integrity of plant membranes, impede signal transduction, and impair stress resistance ability in plants [73,74]. The up-regulation of malonic acid can enhance the resistance of plants to some biotic and abiotic stresses [75,76]. However, as a competitive inhibitor of succinic dehydrogenase, it also hinders the conversion of succinic acid to fumaric acid, thereby disturbing the TCA cycle [77]. The decrease in palmitic acid content and the increase in malonic acid content may be among the factors underpinning the pathogenesis of pod disease in four-seeded vetches. Kaempferol, characterized by its antioxidant and anti-inflammatory properties [78,79], when significantly down-regulated, is likely to reduce the resistance of plants to stress. Quercetin exhibits antioxidant, anti-inflammatory, and antibacterial activities, and its significant up-regulation facilities these functions [80,81]. Luo et al. reported that quercetin inhibited the growth of bacteria such as *Bacillus subtilis* and *Staphylococcus aureus*, as well as fungi such as *Aspergillus niger* and *Canidia albicans* in okra (*Abelmoschus esculentus* L.) to enhance their resistance to stress [82]. It is worth noting that quercetin also favors the colonization of diazotrophic bacteria and mycorrhizal fungi and promotes the growth of root nodules [83,84]. Therefore, the reduction in quercetin may destroy the symbiosis between four-seeded vetches and rhizobia, consequently affecting the physiological process of plants. Thus, four-seeded vetches may also regulate their antioxidant, disease resistance, or antibacterial capacity by inhibiting the expression of kaempferol and quercetin, making it more sensitive to environmental stress.

### 3.5. Analysis of Fumonisin B1 Accumulation and F. proliferatum Pathogenicity in Four-Seeded Vetches

In particular, *F. proliferatum* is one of the main fungi capable of producing fumonisin B1 (FB1) [85]. FB1, as a mycotoxin reducing the disease resistance of plants, can be generated in diverse crops such as rice (*Oryza sativa*) and sugarcane (*Saccharum officinarum*) [86,87,88]. An Italian study on maize (*Zea mays*) confirmed that the pathogenicity of the strain towards plants might not be related to its ability to synthesize fumonisins [89]. This indicates that the pathogenicity of *F. proliferatum* was influenced by multiple factors, such as the presence or absence of certain virulence genes [90]. Metabolomics analysis in this study found that FB1 accumulation in diseased plants was up-regulated. However, the up-regulation of FB1 expression in diseased plants was not significant, which might weaken the credibility of the pathogenicity evaluation, but the pathogenicity of *F. proliferatum* may not depend on its ability to synthesize fumonisins. Thus, the results of the pathogenicity evaluation are still reasonable. In view of the fact that FB1 has been proven to cause a variety of animal diseases [91], exploring strategies to reduce the FB1 level in four-seed vetches in the future may be one of the more effective methods of preventing and controlling its diseases, improving the crop yield, and enhancing the feeding value.

The accumulation of FB1 in plants has complex effects on plant–pathogen interactions and host resistance. The mechanisms of FB1 toxicity involve sphingolipid metabolism, apoptosis, and oxidative stress [92,93]. FB1 can disrupt the normal physiological processes of plants, such as by interfering with membrane integrity and signal transduction, thereby weakening the resistance of hosts and facilitating the invasion and spread of pathogens [94]. Moreover, some defense responses of plants are also triggered by FB1, including the activation of the antioxidant system and the synthesis of defense-related metabolites [95]. At present, the toxicity mechanism of FB1 has not been fully clarified. In the future, understanding these mechanisms is crucial for formulating effective strategies for managing the pod disease of the four-seeded vetch induced by *F. proliferatum*, as well as for reducing the impact of mycotoxin contamination on food safety and crop production.

## 4. Materials and Methods

### 4.1. Sample Collection and Strain Isolation

On 11 April 2024, the infected four-seeded vetches were observed and sampled in a total of three four-seeded vetch growing areas, Rongchang District, Chongqing, China (105°35′ E, 29°24′ N; 105°58′ E, 29°40′ N; 105°60′ E, 29°40′ N). The temperature range in the region is from 20 to 30 °C, with high temperatures and rainy summers. According to the field survey, the average incidence rate in all areas was as high as 92%. The infected pods were collected and treated as follows: thoroughly washed with sterile water, sterilized in 75% ethanol for 30 s, washed with sterile water, then immersed in 1% sodium hypochlorite for 3 min, followed by further washing with sterile water, then they were wiped dry. Finally, the samples were placed on PDA plates and incubated at 28 °C for 7 d in the dark. After that, the colonies were grouped according to the morphological characteristics and purified until a single colony was obtained. The isolate was deposited in the Grassland Microbiology Laboratory, College of Animal Science and Technology, Southwestern University. The colony characteristics were recorded, and the morphological features of all isolates were observed under a microscope (Chongqing UOP Photoelectric Technology Co., Chongqing, China), with representative strains being selected for subsequent experiments.

### 4.2. Pathogenicity Test

The ex vivo experiment: Healthy pods were sterilized in 75% ethanol, punctured with a sterile needle, and inoculated with the mycelia into the wounds. Subsequently, the pods were placed in Petri dishes lined with filter paper and moistened with purified water. Healthy pods that were picked but not inoculated with the pathogenic fungus were taken as controls.

The in vivo experiment: The conidial suspension of the pathogenic fungus with a concentration of 1 × 10^6^ conidia/mL were prepared. Healthy four-seeded vetches with consistent growth in the field were selected. A part of the pods was washed with sterile water and pricked with a sterile needle then sprayed with 20 µL of conidial suspension. Healthy plants sprayed with sterile water served as controls.

All samples were cultured at room temperature; then, the incidence was observed and recorded. The pathogenic fungus was re-isolated from the diseased tissues and compared with those obtained from the previous purification. The isolates which were confirmed to fulfill Koch’s postulates were selected for further experiments.

### 4.3. Morphological and Molecular Identification of the FVS1 Strain

The fungal isolate was cultured on PDA; then, the morphology, color, and diameter of the colony were recorded. By making water slides, the microscopic features of the fungus were examined under a microscope, facilitating the identification of both the species and genera of the strain.

According to the method described by the Fungi Genomic DNA Extraction Kit (Sobolite Technologies Ltd., Beijing, China), the genomic DNA of the fungus was extracted. PCR amplification was conducted using universal primers ITS, ACT, BT, and EF. The primer sequences are presented in Table 3. The reaction system consisted of 0.5 µL of upstream primers, 0.5 µL of downstream primers, 1 µL of DNA template, 12.5 µL of 2 × PCR TaqMaster Mix, and 10.5 µL of ultrapure water. The PCR amplification program was as follows: pre-denaturation at 94 °C for 5 min, followed by 35 cycles of denaturation at 94 °C for 30 s, annealing at 54 °C for 30 s, extension at 72 °C for 90 s, and finally, extension at 72 °C for 10 min. The amplification products were sent to GENEWIZ (Suzhou, China) for sequencing. The resulting sequences were uploaded to the NCBI database for BLAST similarity comparison; sequences exhibiting high similarity were downloaded for phylogenetic analysis, and a congruence test was performed prior to concatenating the loci. A phylogenetic tree was constructed using the NJ method, and *p*-distance was implemented in MEGA11 software, with 1000 bootstrap replications.

### 4.4. Growth of FVS1 Strain in Different Media

The utilization of carbon and nitrogen sources by fungi varied. In this study, five different media were selected for experimentation. Under 24 h darkness (24 hD/0 hL), the strain was inoculated on PDA, SCDA, PSA, MEA, and OA at 28 °C. After 3 d and 7 d inoculation, the colony diameter was measured using the crisscross method [96], while spore production in different media was assessed via the hematological counting plate method [97]. Each treatment was repeated six times.

### 4.5. Growth of FVS1 Strain Under Different Temperature Conditions

The mycelia were inoculated onto PDA medium and cultured at 5 °C, 10 °C, 15 °C, 20 °C, 25 °C, 30 °C, and 35 °C in darkness. After 3 d and 7 d, the colony diameter and spore production were measured. Six replications were performed for each treatment.

### 4.6. Growth of FVS1 Strain Under Different Light Durations

The fungal strain was cultured on PDA at 28 °C, with photoperiods of continuous darkness (24 hD/0 hL), continuous light (0 hD/24 hL), and 12 h of light and 12 h of darkness (12 hD/12 hL). The light intensity was set to 2000 lx. The colony diameter and spore production were quantified after 3 and 7 d. Each treatment had six replicates.

### 4.7. Growth of FVS1 Strain Under Different pH Conditions

PDA plates with pH values adjusted to 5, 7, 9, and 11 were prepared, and mycelia were inoculated onto the medium and incubated under full darkness at 28 °C. The colony diameter and spore production were determined after 3 d and 7 d, with six replications being set up for each group.

### 4.8. Analysis of Metabolites

Samples from diseased plants (B) and healthy plants (CK) were dried in a freeze dryer (Beijing Songyuan Huaxing Co., Beijing, China) for 24 h and then grounded into powder using a high-throughput tissue grinder (Ningbo Xinzhi Biotechnology Co., Ningbo, China) at 50 Hz for 10 min. Next, 50 mg of the powder from each group was transferred to EP tubes. Then, 0.6 mL of 70% methanol was added to each tube, and the samples were left to stand at 4 °C for 12 h, followed by ultrasonication for 5 min under 25–40 KHz and centrifugation at 10,000 r/min for 10 min. The supernatant was collected. Each group had six biological replicates. To ensure data reliability, quality control (QC) samples were prepared by mixing 10 μL of samples from B group and CK group, while one QC sample was interspersed in every six samples. LC-MS/MS analysis was carried out using ultra-high-performance liquid chromatography (UHPLC) UltiMate 3000 (Dionex, Sunnyvale, CA, USA), following the previous loading method used by the research group for metabolomics samples [98].

### 4.9. Screening of Biocontrol Fungi

Using the plate confrontation method [99], FVS1 was inoculated in the center of one side of the PDA, while the center of the other side was inoculated with biocontrol fungi. PDA plates inoculated only with FVS1 were used as the control group. The fungal strains with high inhibitory activity, having been screened by the research group, were selected for the experiment. The fungi used were *Trichoderma harzianum*-M1 (PV276763), *T. harzianum*-M2 (PV2767634), *T. harzianum*-M3 (PV276992), and *T. harzianum*-MM (PV276991). Each treatment was replicated six times. All plates were incubated at 28 °C in the dark. After 3 d and 7 d, the colony diameter was measured, and the rate of growth inhibition was calculated.

Rate of growth inhibition (%) = (colony diameter of control − colony diameter of treatment)/colony diameter of control × 100% [100].

### 4.10. Statistical Analysis

The experimental data regarding the physiological characteristics of the pathogenic fungus and the inhibition rate of biocontrol fungi were statistically analyzed via one-way analysis of variance (ANOVA) using Excel 2021 and SPSS 30.0. A Student–Newman–Keuls (S-N-K) post hoc test was applied, with statistical significance set at *p* < 0.05. For metabolomics data, they were imported into Compound Discoverer 3.3 and matched against the Mz Cloud and Mz Vault databases after peak detection, extraction, deconvolution, normalization, and peak alignment. Principal component analysis (PCA) of all samples was carried out on MetWare Cloud (https://cloud.metware.cn, accessed on 27 January 2025), a free online data analysis platform, to generate PCA score maps. Differential metabolites were screened based on variable importance in terms of projection (VIP > 1) and significance level (*p* < 0.05), and volcanic plots and heat maps were created. In order to understand the position of various metabolites in the metabolic network and to identify the key metabolic pathways, the screened differential metabolites were analyzed by means of MetaboAnalyst 6.0 (https://www.metaboanalyst.ca/, accessed on 31 January 2025). Moreover, KEGG database was utilized to annotate metabolic pathways; then, the pathways were mapped using PowerPoint 2021.

## 5. Conclusions

In this study, it is first confirmed that *F. proliferatum* is the main pathogen causing pod disease in four-seed vetches. The growth of this fungus is affected by nutritional conditions and the external environment, but it has strong environmental adaptability. The metabolomics analysis revealed that *F. proliferatum* would reduce the stress resistance of four-seed vetches, which might be achieved by significantly down-regulating palmitic acid, kaempferol, and quercetin and up-regulating malonic acid. Furthermore, the four-seeded vetch may resist the invasion of the pathogen by regulating lipid metabolism, amino acid metabolism, and flavonoid biosynthesis, such as via the accumulation of histidine, naringenin, and catechin in infected plants. *T. harzianum* showed strong inhibition of the growth of *F. proliferatum*, and the most effective strain for control was M3. The results of the identification, pathogenicity test, biological characterization, metabolomics analysis, and biocontrol fungi screening of the pathogenic fungus on the four-seeded vetches not only provide valuable information for further understanding the disease process but also open new paths for its integrated control and for increasing crop yield.

## Figures and Tables

**Table 1:** Details of the strains used for phylogenetic analysis.

Species	Accession Number ^1,2,3^	Locality	Substrate	GenBank Accession Numbers ^4^	
				TUB2	TEF1
*F. proliferatum*	-	Malaysia	*Hylocereus polyrhizus*	JX868893	JX868976
*F. proliferatum*	NRRL 66417	USA	*Vitis vinifera*	MH398182	KX656215
*F. proliferatum*	NRRL 66451	USA	*V*. *vinifera*	MH398240	KX656221
*F. anthophilum*	CBS 136.95	Netherlands	*Amaryllis*	MW402257	MW402058
*F. fujikuroi*	CBS 240.64	Japan	*Oryza sativa*	MW402315	MW402117
*F. verticillioides*	CBS 139.40	Italy	*Phyllocactus hybridus*	MW402263	MW402064
*F. concentricum*	CBS 453.97	Guatemala	*Musa sapientum*	MN534123	MN533998
*F. ananatum*	CMW 28599	South Africa	*Ananas comosus*	MW402358	MW402157
*F. globosum*	CBS 120992	South Africa	*Maize kernels*	MW402198	MW401998
*F. madaense*	CBS 146651	Nigeria	*Sorghum*	MW402295	MW402096
*F. sterilihyposum*	NRRL 53991	Brazil	*Mangifera indica*	GU737305	GU737413
*F. sacchari*	CBS 183.32	-	*Saccharum officinarum*	MW402302	MW402104
*F. nygamai*	CBS 120995	Australia	*Sorghum root*	MW402199	MW401999
*F. tupiense*	NRRL 53996	Brazil	*Mangifera indica*	DQ445782	DQ452860
*F. lactis*	CBS 420.97	USA	*Ficus carica*	MN534078	MN534015
*F.pseudonygamai*	CBS 416.97	Nigeria	*Pennisetum typhoides*	MN534064	MN534030

^1^ CBS = Culture Collection of the Westerdijk Fungal Biodiversity Institute, Utrecht, the Netherlands; ^2^ CMW = Culture Collection at the FABI, University of Pretoria, South Africa; ^3^ NRRL = Agricultural Research Service Culture Collection, USA; ^4^ TUB2 = beta-tubulin; TEF1 = translation elongation factor-1alpha gene.

**Table 2:** The sporulation amounts of FVS1 under different conditions.

Item	Treatments	FVS1
Different media	PDA	6.865 ± 0.107 ^a^
	SCDA	6.157 ± 0.135 ^c^
	PSA	6.734 ± 0.079 ^ab^
	MEA	6.554 ± 0.222 ^b^
	OA	6.769 ± 0.096 ^ab^
Different temperature (℃)	5	-
	10	-
	15	6.037 ± 0.252 ^b^
	20	6.278 ± 0.165 ^ab^
	25	6.518 ± 0.165 ^a^
	30	6.458 ± 0.135 ^a^
	35	-
Different light durations (h)	0 h/24	6.614 ± 0.210 ^a^
	24 h/0	6.649 ± 0.240 ^a^
	12 h/12	6.579 ± 0.165 ^a^
Different pH	5	6.950 ± 0.068 ^ab^
	7	7.070 ± 0.096 ^a^
	9	6.830 ± 0.130 ^b^
	11	6.278 ± 0.165 ^c^

Note: The data in the table represent the number of spores after lg conversion. The same lowercase letters mean the difference is not significant (*p* > 0.05); different lowercase letters indicate significant differences (*p* < 0.05). “-” indicates that no spores are produced under these conditions.

**Table 3:** Sequences of the primers.

Gene ^1^	Primers	Sequence (5′-3′)	References
ITS	ITS1	TCCGTAGGTGAACCTGCGG	[19]
	ITS4	TCCTCCGCTTATTGATATGC	
ACT	ACT512F	ATGTGCAAGGCCGGTTTCGC	[20]
	ACT783R	TACGAGTCCTTCTGGCCCAT	
Tub	BT-2a	GGTAACCAAATCGGTGCTGCTTTC	[21]
	BT-2b	ACCCTCAGTGTAGTGACCCTTGGC	
Tef	EF1-728F	CATCGAGAAGTTCGAGAAGG	[22,23]
	EF-2	GGARGTACCAGTSATCATGTT	

^1^ ITS = internal transcribed spacers and intervening 5.8S nrDNA; ACT = partial actin gene.

## Data Availability

Data are contained within the article.
